# Advancing Inclusivity for African American and Hispanic Populations in Alzheimer’s Disease Trials: Understanding Screen Failures from the Aducanumab Confirmatory Trial

**DOI:** 10.1002/alz.095664

**Published:** 2025-01-09

**Authors:** Shardae Showell, Jennifer Murphy, Gersham Dent, Katherine Dawson

**Affiliations:** ^1^ Biogen, Cambridge, MA USA

## Abstract

**Background:**

Given the high prevalence of Alzheimer’s disease (AD) and historic underrepresentation of African Americans and Hispanics in AD clinical trials, there is a critical need to advance understanding of inclusive clinical trial considerations. ENVISION was a confirmatory study designed to validate clinical efficacy of aducanumab, which included specific goals for African American and Hispanic population enrollment in the United States. In our pursuit, we found a 90% screen failure rate among African Americans and Hispanics compared to 75% in non‐Hispanic whites. In this analysis, the documented reasons for screen failure among African American and Hispanic participants were evaluated. These data are shared to foster a dialogue that encourages the development of more inclusive trial design in future AD studies.

**Method:**

ENVISION (NCT05310071) was a multicenter, randomized, double‐blind, placebo‐controlled, phase 3b/4 confirmatory study of aducanumab in participants with mild cognitive impairment (MCI) and mild dementia AD. Data from the ENVISION screen failure population of African Americans (N = 75) and Hispanics (N = 110) within the United States were analyzed, and reasons for screening failure based on inclusion‐exclusion criteria and health characteristics are reported.

**Result:**

Not meeting the inclusion requirement for MCI or mild AD according to NIA‐AA criteria was the most common reason for screen failure at 55% and 58%, for African Americans and Hispanics respectively. The remaining causes of screen failures include unconfirmed amyloid beta pathology (African Americans, Hispanics: 7%, 14%), brain MRI exclusionary findings (1%, 6%), and the lack of an identified care partner (3%,1%). However unspecified reasons in the opinion of the investigator represent 19% of African Americans and 8% of Hispanics. Assessment of baseline physical health characteristics known to be AD risk factors revealed a higher incidence of elevated levels of hemoglobin A1c and blood pressure among the screen failed population of African Americans and Hispanics compared to randomized participants.

**Conclusion:**

The screen failed population of African Americans and Hispanics represent a group of individuals that are eager to participate in AD clinical trials, but unable to. This hypothesis generating analysis provides insight for new avenues of future exploration in clinical trial design for better inclusion of underrepresented populations.

